# Multiple aneurysms coexisting with carotid occlusion revealed by cerebral infarction: A case report

**DOI:** 10.1016/j.radcr.2022.07.116

**Published:** 2022-08-29

**Authors:** Ahmadou Bamba Mbodji, Ibrahima Faye, Ibrahima Diassé, Abdoulaye Ndoye Diop

**Affiliations:** a Fann University Hospital, Dakar, Sénégal; b Ledantec University Hospital, Dakar, Sénégal; c Gaston Berger University of Saint-Louis, Saint-Louis, Sénégal

**Keywords:** Intracranial aneurysm, Carotid arteries, Cerebral infarction, CT angiogram of supra-aortic arteries

## Abstract

Intracranial aneurysms are focal dilations of an intracranial artery. They can be discovered incidentally, during a hemorrhagic stroke or subarachnoid hemorrhage, but it is rare for it to be detected after an ischemic stroke. The prevalence of the association between symptomatic carotid occlusion or stenosis and intracranial aneurysms is estimated to be 6.3%. We report the case of a patient hospitalized for the management of a stroke in whom investigations had revealed the coexistence of right carotid occlusion and multiple aneurysms in the right middle cerebral artery. The diagnosis was made by CT angiography of supra-aortic trunks.

## Introduction

Cerebral aneurysms are abnormal focal pouch-like dilatations of the cerebral artery. They may be revealed incidentally [1] or after an hemorrhagic stroke, but can exceptionally be revealed by carotid occlusion and/or stenosis [2,3]. We report the case of a 70-year-old patient with multiple intracranial aneurysms of the right middle cerebral artery associated with ipsilateral internal carotid artery occlusion and contralateral internal carotid artery stenosis.

The prevalence of combined carotid occlusion or stenosis and intracranial aneurysm is estimated at 6.3% [3]. This value included both intracranial aneurysms ipsilateral and contralateral to internal carotid artery stenosis. In the meta-analysis by Werner [8], only 150 documented cases were reported in the current literature. The mechanism of aneurysm occurrence, although discussed, is thought to be related to intracranial hemodynamic changes.

## Case report

This is the case of a 70-year-old hypertensive patient on Amlodipine 10 mg for 1 year with poor compliance. He presented with left hemiplegia associated with a speech disorder of abrupt onset evolving for about 15 days. The clinical examination on admission revealed a capsular left hemiplegia, a comprehensible dysarthria, an auscultatory arrhythmia, and a NIHSS score of 12.

Laboratory tests revealed a normocytic anemia with a hemoglobin level of 9.5 g/dL, the platelet count was 443,000/mm^3^. The blood count was normal with a prothrombin rate (PT) of 75% and an\International Normalized Ratio (INR) of 1.28.

**Fig. 1:**
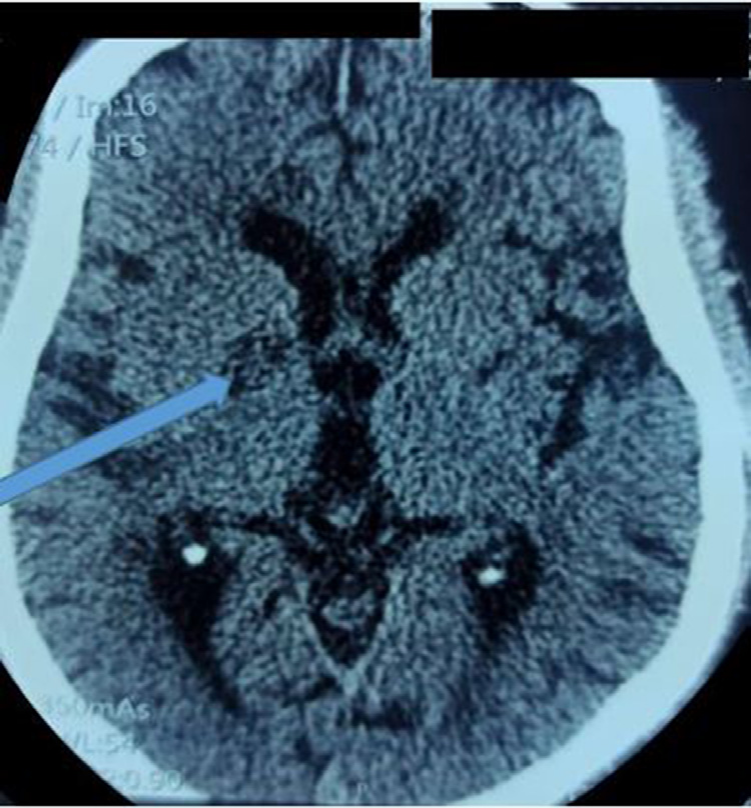
Axial section of brain CT scan without injection showing a right internal capsular infarct.

A cerebral CT scan without contrast showed a semi-focal infarction in the right superficial sylvian territory and a sequential infarction in the left deep sylvian territory (Fig. 1). The electrocardiogram (EKG) showed atrial fibrillation associated with atrial extrasystoles, but the echocardiogram was unremarkable.

**Fig. 2:**
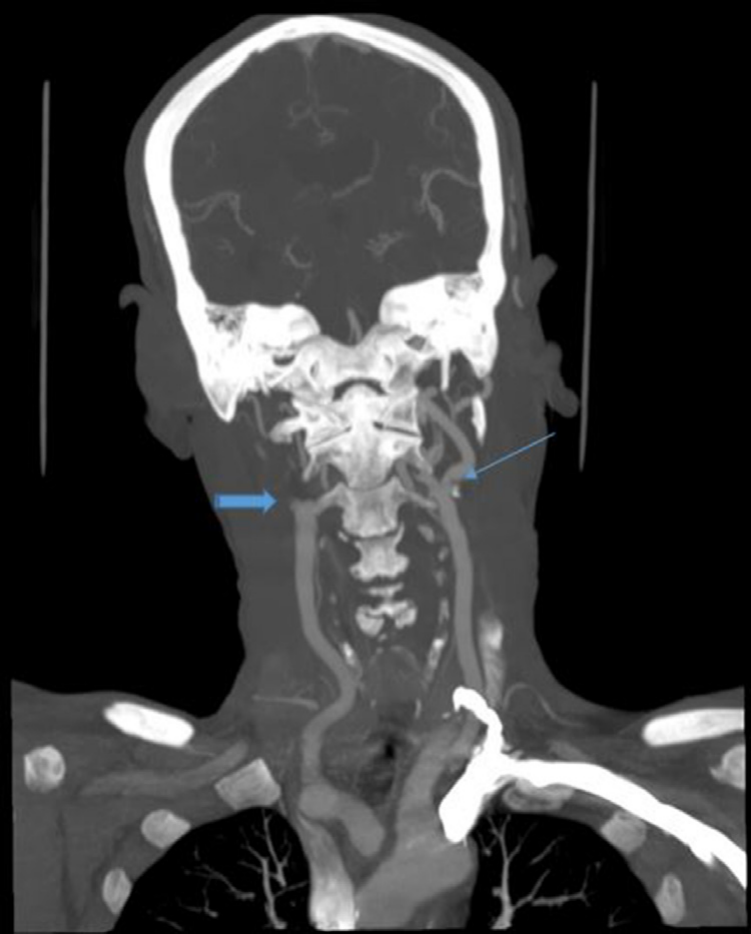
Supra-aortic arteries CT scan showed an occlusion of the right internal carotid artery (striped right arrow) and left bulbar.

**Fig. 3:**
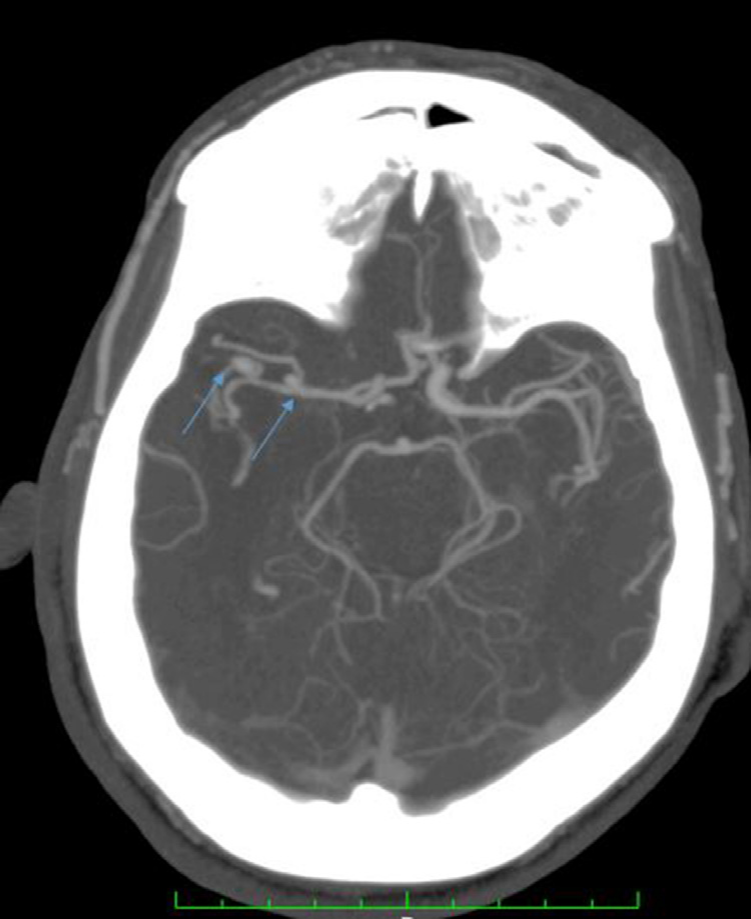
Axial section of cerebral CT scan in MIP (maximum intensity projection) reconstruction showing 2 aneurysms in the right M1 branch (arrows).

A CT angiogram of supra-aortic arteries showed complete occlusion of the right internal carotid artery from its origin (Fig. 2), associated with 2 unruptured arterial aneurysms in the M1 portion of the ipsilateral middle cerebral artery measuring 5.25 × 4.2 mm and 4.17 × 2.67 mm with vessel size reduction (Fig. 3).

On the left side, a mixed stenosing bulbar plaque of more than 50% was noted (Fig. 2).

The diagnosis of hyperflow aneurysms was evoked. Our patient was treated with beta-blockers, preventive anticoagulation, statins and antihypertensive drugs.

The patient's evolution was unfavorable marked by a death due to complications of decubitus before a neurosurgical intervention. Unfortunately the autopsy was not performed.

## Discussion

The discovery of intracranial aneurysms during the etiological investigation of a cerebral infarction is an uncommon situation in routine clinical practice ([4], [5], [6]]. Our patient had 2 unruptured intracranial aneurysms of the right middle cerebral artery associated with an ipsilateral occlusion of the internal carotid artery. The frequency of association of carotid occlusion or stenosis with the intracranial aneurysm is estimated to be 6.3% [3]. This is more than twice the prevalence of intracranial aneurysms in the general population, which is estimated at 2.8% [4,7]. According to a meta-analysis by Werner [8] only 150 documented cases have been reported in the current literature. Of those, 134 (89.3%) had a single aneurysm and 16 (10.7%) had multiple aneurysms [3]. The pathophysiology of intracranial aneurysm formation during carotid occlusion or stenosis is not well understood. In fact, hemodynamic changes caused by carotid stenosis could lead to the appearance of intracranial aneurysms. It is believed that ipsilateral stenosis aneurysms are linked to increased flow and speed of blood circulation, which exert stress on the walls of the arterial vascular system, and change the architecture of the vascular wall. The middle cerebral artery is the preferential area for the occurrence of these multiple aneurysms, as found in our patient [8]. The existence of multiple aneurysms in our patient could also be explained either by ipsilateral carotid occlusion or by contralateral internal carotid stenosis. However, this last hypothesis seems less likely because aneurysms due to contralateral carotid stenosis are more likely to be located in communicating branches [9].

## Conclusion

The association between intracranial aneurysm and carotid occlusion or stenosis is a rare situation in clinical practice which mechanisms of occurrence are unknown. Management is poorly codified and requires multidisciplinary collaboration. The CT angiogram of supra-aortic arteries is the key examination to evoke the diagnosis of hyperflow aneurysms, hence its interest in the etiological research of ischemic stroke.
